# Parental knowledge and practices on infant teething, Taif, Saudi Arabia

**DOI:** 10.1186/s13104-015-1690-y

**Published:** 2015-11-23

**Authors:** Abubaker Ibrahim Elbur, M. A. Yousif, Ahmed Abdulrahman Albarraq, Mustafa A. Abdallah

**Affiliations:** Pharmacy Practice Research Unit, College of Pharmacy, Taif University, Kingdom of Saudi Arabia Al-Haweeiah, P. O. Box 888, Taif, 21974 Kingdom of Saudi Arabia; PTRC, Academic Affairs and Training, Armed Forces Hospitals, Taif Region, Taif, Kingdom of Saudi Arabia

**Keywords:** Parents, Knowledge, Practices, Infant teething, Saudi Arabia

## Abstract

**Background:**

Parents’ false beliefs about signs and symptoms associated with teething have been documented in many studies around the world. This study was conducted to assess parental knowledge on infant teething process and to investigate parents’ practices used to alleviate teething disturbances.

**Methods:**

A cross-sectional survey was conducted among parents of children of 6 months–5 years old in Taif, Saudi Arabia during April 2013. Convenience method of sampling was adopted and the data was collected by mean of a structured-questionnaire. Data was processed by SPPS. Logistic regression analysis was performed. P value <0.05 was considered statistically significant.

**Results:**

Overall, of 493 participants were included in the final analysis with mean age 35 years. Females constituted more than two-third. All the parents attributed one or more of the listed signs and symptoms to teething process. Desire to bite, fever, gum irritation, increased salivation and diarrhea were the most reported signs and symptoms of teething by 459 (93.1 %), 429 (87 %), 415 (84.2 %), 414 (84 %) and 409 (83 %) of the parents respectively. The only predictor of ascribing fever as a sign of infant teething was female gender (P = 0.001). However, female gender (P < 0.001), residence (P = 0.039) and educational level (P = 0.006) were found to be significantly associated with ascribing diarrhea as one of the teething symptoms. Only 91 (18.5 %) of the parents responded correctly to all questions designed to assess their knowledge on teething process.

**Conclusions:**

Wide gaps in parents’ knowledge and practices related infant teething was identified. Educational interventions are needed to upgrade parents’ knowledge and improve their practices regarding infant teething process.

## Background

Teething is a natural physiological process that all children experience and generally commences from 6 months to about 3 years of age [1]. The appearance of the first tooth in the oral cavity of an infant is considered as an important milestone in the child’s life.

A broad range of symptoms may occur concomitantly with teething. However, no scientific evidence is available to suggest that there are any symptoms or signs specific to teething [2]. The association between primary tooth eruption and minor symptoms like irritability, increased salivation, runny nose, loss of appetite, diarrhea, rash, and sleep disturbance was reported [3]. However, severe signs and symptoms such as fever were not documented. The incidence of mild symptoms that are temporally associated with primary tooth eruption may be in part a consequence of the change from a passive to an active immune system [4].

Parents have false beliefs about signs and symptoms associated with teething [5–9]. The commonest medical problems ascribed by parents were fever [5, 9] and diarrhea [6, 10]. The consequence of such misconceptions is that the incidence of such symptoms may be signs of an underlying serious condition which may endanger the life of the child.

The perception of teething problems was found to be significantly associated with parents’ educational level [11]. Generally, professionals and parents with high income were found to have a better level of knowledge about teething [10].

Different pharmacological and non-pharmacological strategies are usually used as teething remedies without the advice of a dental professional [12]. Parents commonly used systemic and topical analgesics to relieve teething pain [9] and even antibiotics to treat associated symptoms [13]. In addition, parents allow their children to bite on chilled objects to relieve symptoms associated with teething [14].

To our knowledge, no attempt had previously been made in Saudi Arabia to assess parental knowledge and practices on infant teething process. Therefore, the present study was undertaken to assess parental knowledge on infant teething and to investigate parents’ practices used to alleviate teething disturbances. Furthermore, it is expected that the findings of the study will help globally or at least at the regional level in the design of educational messages to upgrade parental knowledge and improve their practices on infant teething process.

## Methods

### Study design and study area

A cross-sectional survey was conducted in Taif Area, Kingdom of Saudi Arabia during the period of 1 month (April 2013).

### Inclusion and exclusion criteria

Parents who admitted that they were fathers or mothers of children of 6 months to 5 years old were invited to participate in the survey. The exclusion criteria were: parents of children of age >5 year old, those who refused to participate in the study and who were mentally incapable to communicate. If both parents participated, their responses were recorded in a separate data collection tool. The objectives of the study were stated clearly for the parents before commencement of the interview process.

### Sample size and sampling technique

A convenience method of sampling was adopted to recruit the parents. Based on the research team experience on such types of studies in the specified study area, a sample of 500 parents was considered to be sufficient. Of note, a post hoc power calculation was conducted to determine if this sample was really representing the study population. Based on the last census conducted in the year 2010 in the country [15], the total number of parents in the study area was estimated to be 1,200,000. Based on that census, the sample size was calculated to be 384. Sample calculation was conducted at a 95 % confidence level with a margin of error 5 %. Considering the population growth the actual number of the included participants was sufficient to represent the parents in the study area.

### Data collection

Data was collected by ten trained third year pharmacy students (males and females) as part their training activities in the Research Methodology subject. The data was collected in public places in the city (Malls, supermarkets, parks, schools, and restaurants). Some students interviewed their closed relatives at homes. The students used face-to face- interview method to gather the data. The average time to conduct the interview was estimated to be 10 min. For the purpose of data collection a 17-item, structured-questionnaire was designed after thoroughly searching the relevant literature. The items included in the questionnaire were adapted from those used in a previous study [10] with slight modifications. The questions were distributed through four sections. Part one was designed to collect data on parents’ demographics (gender, age in year, residence and level of education). The second part was designed to collect data on signs and symptoms the parent perceived to be associated with teething. In this respect, 14 signs and symptoms were recorded based on those ascribed by parents in similar previous studies. A third part was designed to assess parents’ knowledge on teething process through four questions. The last part composed of questions to investigate parents’ practices used to relieve pain and to treat disturbances attributed to teething (the use of systemic and topical analgesics and chilled objects to relieve pain). The questionnaire was tested with a group of ten parents. Minor observations were suggested and consequently adopted in the final questionnaire.

### Data analysis

Data was processed using the software SPPS (21.0 SPSS Inc., Chicago IL, USA). Descriptive statistics were used to describe all variables. Logistic regression analysis was performed to determine the most significant demographic variables (independent) associated with reporting fever and diarrhea (dependent) as signs and symptoms of infant teething. Crude logistic regression analyses were performed as initial steps of qualifying covariates to be included in multivariate logistic regression analyses. P value <0.05 was considered statistically significant.

### Ethical approval

Ethical approval for the conduction of the research was obtained from the Pharmacy Practice Research Unit (PPRU), College of Pharmacy, Taif University, Saudi Arabia.

## Results

### Parents’ demographics

Overall, 510 parents agreed to participate. However, data from 493 (96.7 %) participants were included in the final analysis, while 17 (3.3 %) were rejected due to missing of vital data (parents apologized to complete the questionnaire after initially agreed to participate). Females constituted more than two-third of parents. Mean age was 35 years. Town dwellers were 433 (87.8 %) and 308 (62.5 %) had university education. Out of the university graduates 280 (64.7 %) were living in the city, while 28 (46.7 %) were residing outside the town, (P < 0.001). Educational level was found to be inversely proportional to parents’ age (P < 0.001). Table 1 showed the demographic characteristics of the participants.

**Table 1:** Parents’ demographic characteristics

Background characteristic	Frequency	Percentage
Gender		
Male	156	31.6
Female	337	68.4
Age groups (in year)		
<35	278	56.4
>35	215	43.6
Residence		
Town	433	87.8
Outside town	60	12.2
Educational level		
University and above	308	62.5
Secondary	135	27.4
Primary	37	7.5
Illiterate	13	2.6
Total	493	100

### Teething signs and symptoms

Overall, 100 % of the parents attributed one or more of the listed signs and symptoms to teething process. Desire to bite, fever, gum irritation, increased salivation and diarrhea were the most reported signs and symptoms of teething by 459 (93.1 %), 429 (87 %), 415 (84.2 %), 414 (84 %) and 409 (83 %) of the parents respectively. All signs and symptoms attributed to the infant teething process as disclosed by parents were presented in Table 2.

**Table 2:** Signs and symptoms of teething as disclosed by parents

Sign/symptom	Agree	Disagree	Don’t know
Fever	429 (87 %)	45 (9.1 %)	19 (3.9 %)
Diarrhoea	409 (83 %)	70 (14.2 %)	14 (2.8 %)
Sleep disturbance/wakefulness	364 (73.8 %)	78 (15.8 %)	51 (10.3 %)
Loss of appetite	377 (76.5 %)	73 (14.8 %)	43 (8.7 %)
Gum irritation	415 (84.2 %)	38 (7.7 %)	40 (8.1 %)
Desire to bite	459 (93.1 %)	22 (4.5 %)	12 (2.4 %)
Increased salivation	414 (84 %)	49 (9.9 %)	30 (6.1 %)
Runny nose	172 (34.9 %)	247 (50.1 %)	74 (15 %)
Respiratory system problems	105 (21.3 %)	289 (58.6 %)	99 (20.1 %)
Skin rash	65 (13.2 %)	350 (71 %)	78 (15.8 %)
Vomiting	236 (47.9 %)	198 (40.2 %)	59 (12 %)
Ear problems	176 (35.7 %)	215 (43.6 %)	102 (20.7 %)
Convulsions	61 (12.4 %)	361 (73.2 %)	71 (14.4 %)
Increased susceptibility to other diseases	189 (38.3 %)	187 (37.9 %)	117 (23.7 %)

The results of regression analysis were presented in Tables 3 and 4. The only predictor of ascribing fever as a sign of infant teething was female gender (P = 0.001). However, female gender (P < 0.001), residence (P = 0.039) and educational level (P = 0.006) were found to be significantly associated with ascribing diarrhea as one of the teething symptoms.

**Table 3:** Predictors of reporting fever as a sign of infant teething

Covariates	% of agree	n	Univariable analysis crude OR(95 % CL)	P value	Multivariable analysis adjusted OR(95 % CL)	P value
Gender						
Male	78.8	156	0.4 (0.2–0.6)	<0.001	0.4 (0.2–0.7)	0.001
Female	90.8	337				
Age group in year						
<35	87.1	278	1.0 (0.6–1.7)	0.981		
>35	87.0	215				
Residence						
Outside city	76.7	60	2.3 (1.2–4.5)	0.013		
City	88.5	433				
Educational level						
Below university	88.1	185	0.9 (0.5–1.5)	0.577		
University	86.4	308				
Total	493

**Table 4:** Predictors of reporting diarrhea as a symptom of infant teething

Covariates	% of agree	n	Univariable analysis crude OR(95 % CI)	P value	Multivariable analysis adjusted OR(95 % CL)	P value
Gender						
Male	70.5	156	0.3 (0.2–0.5)	<0.001	0.3 (0.2–0.5)	<0.001
Female	88.7	337				
Age group in year						
<35	79.9	278	0.6 (0.4–1.0)	0.038		
>35	87.0	215				
Residence						
Outside city	71.7	60	2.2 (1.7–4.0)	0.015	2.0 (1.0–3.9)	0.039
City	84.5	438				
Educational level						
University	79.5	308	0.5 (0.3–0.8)	0.010	0.5 (0.3–0.8)	0.006
Below university	88.6	185				
Total	493					

### Parental knowledge on teething

Of all interviewed parents 383 (77.7 %) agreed that babes’ teeth start to erupt around 6–7 months of age and 404 (81.9 %) believed that the first teeth to appear in the mouth are the lower central incisors. More than two-third (68.6 %) of the participants agreed with the fact that the eruption of teeth gets completed in approximately 2 years of age and 153 (32.7 %) considered that delayed eruption of teeth may be an indicator of the presence of systemic disease. Overall, only 91 (18.5 %) of the parents responded correctly to all questions designed to assess their knowledge on teething process. In this respect, no single parents’ background characteristic was found to be associated with the overall knowledge on teething process.

### Practices to relive teething pain and management of other teething problems

A considerable number 434 (88 %) of parents agreed to the statement “allowing the child to bite on a chilled object will relieve pain associated with teething”, while 346 (70.2 %) preferred the use of systemic analgesics.

Out of the total parents 451 (91.5 %) agreed that they should consult a physician in case of any problems with tooth eruption, while 222 (45 %) believed that giving antibiotics will relieve symptoms related to teething. Table 5 showed parents’ responses regarding practices to relieve pain and other teething problems.

**Table 5:** Parents’ responses regarding practices to relieve pain and management of other teething problems

Items	Agree	Disagree	Don’t know
Allow the child to bite on a chilled object	434 (88 %)	40 (8.1 %)	19 (3.9 %)
Allow bottle feeding or nursing at night	192 (38.9 %)	163 (33.1 %)	138 (28 %)
Use systemic analgesics	346 (70.2 %)	110 (22.3 %)	37 (7.5 %)
Apply topical analgesics to the gum	333 (67.5 %)	84 (17 %)	76 (15.4 %)
Giving the child fluids to prevent dehydration	432 (87.6 %)	28 (5.7 %)	33 (6.7 %)
Giving antibiotics to relieve symptoms related to teething	222 (45 %)	166 (33.7 %)	105 (21.3 %)
Consultation of physician in case of any problems with tooth eruption	451 (91.5 %)	33 (6.7 %)	9 (1.8 %)

## Discussion

The current study was conducted among parents in Taif city; Saudi Arabia. Nearly 63 % of them attained university level of education. This finding may be attributed to the fact that the majority of the participants was living in the town where they were originally born and had better chances to accomplish this level of education. Moreover, highly educated parents are expected to participate readily in the survey compared to non-educated ones.

Generally, as documented in previous studies the results of the current survey showed that the parents had misconceptions and poor knowledge on infant teething. The participants attributed one or more of the listed signs and symptoms to teething process. A possible explanation of this finding is the influence of the local cultural beliefs beside the false messages delivered to the parents by some healthcare providers. Comparatively, other researchers quoted a lower percentage (64.8 %) in a Nigerian survey conducted among nursing mothers [16].

Fever during the process of primary tooth eruption is caused by human teething virus (HT virus), which at the beginning of life is responsible for primary infection that becomes subclinical [17]. Fever was ascribed by 87 % of the respondents as a symptom associated with teething. As noted in another study conducted in the same study area parents had poor knowledge of determining the threshold for defining fever [18], which may partially explain the higher percentage observed in the current survey. Comparatively, this percentage was higher than the percentages of 71.1 and 70 % reported in two different studies [5] and [10] respectively. In contrast, Utiet al [6] reported slightly higher percentage (90 %) than what was documented in the current study.

Eighty-three percent of the participants attributed diarrhea to teething. In contrast, this percentage was much higher than the percentages of 37.9 and 75 % obtained in two previous studies [9, 14] respectively. Diarrhea was reported in a recent prospective study as one of the symptoms associated with teething [3], but poor personal and environmental hygiene practices may be major contributory factors that increase the incidence of diarrhea among children in this age range.

Only 18.5 % of all the participants responded correctly to all questions designed to assess their knowledge about teething. No single demographic variable was found to be associated with the total knowledge. This percentage was very low compared to the percentage of 71.4 % reported in the above mentioned Indian study [10]. Poor knowledge on such a common public health issue may be highly attributed to the absence of proper health education, which can be better provided through primary health care facilities in the area.

Despite uncertainty about teething pain per se, most parents preferred to manage it using a combination of non- pharmacological and pharmacological treatment [12]. Teething must be treated in the first instance with an appropriate device which applies local pressure to the gingivae. The use of pharmacological treatment can be limited to certain cases with careful monitoring [19]. Approximately of all the recruited parents, 88 % agreed that allowing the child to bite on a chilled object will alleviate pain associated with teething. In contrast, other authors found that 34.3 % of the parents allow their children to chew on objects to relieve pain [20]. Nearly 68 % of the participants agreed to the use of systemic analgesics to relieve teething pain and 66.8 % preferred to apply them topically. In another survey, 61 % of the questioned parents considered the systemic approach to be effective for the management of teething pain [13]. Parents should be educated to rationally use both forms of analgesics as the use of the latter was documented to compromise a child’s life [12].

One of the most serious finding noted in the present study was that 45 % of the parents agreed that antibiotics can be used to treat symptoms and signs associated with teething process. Misuse of antibiotics can harm both the child and the community as it is associated with the emergence of bacterial resistance. The use of antibiotics for treatment of disturbances associated with teething was not reported in developed countries [9]. In Saudi Arabia, as in many other countries, antibiotics are available for sale without restrictions in community pharmacies [21]. Dispensing antibiotics without medical prescription and the false beliefs of the parents about antibiotics may encourage them to self-medicate their children in such conditions. Nearly 92 % of the participants agreed that they should consult a physician in case of any problem related to tooth eruption. Consultation of a physician is a positive finding as it is considered as the first rational step towards the proper management of the child problem. In the above mentioned study [13], 54 % of the parents claimed that consulting a pediatrician was not their immediate first line treatment approach as few symptoms, such as diarrhea, fever, and skin rash could be corrected by using routine analgesic and antibiotics.

This study had some limitations. Firstly, it was conducted in a single town in Saudi Arabia. Therefore, the obtained results cannot be applied to the general population. Future researches on this topic can recruit a larger sample of parents from different areas in the country to deeply investigate parents’ knowledge and practices on teething process. Secondly, the data obtained from the parents of older children specifically responses on teething symptoms was subjected to recall bias.

## Conclusion

In conclusion, the obtained results showed a wide gap in knowledge and misconceptions with regard to the ascribed signs and symptoms and practices used to manage infant teething problems. Educational interventions are needed and should focus on educating the parents to better recognize the signs and symptoms attributed to teething and when and where to seek medical consultation and the proper use of medications to relieve pain or to rationally treat problems associated with teething. The interventions can be better provided through the well-distributed primary health care facilities in the city in a simplified and culturally acceptable way.
